# Formation of Multinucleated Giant Cells after Experimental Intracerebral Hemorrhage: Characteristics and Role of Complement C3

**DOI:** 10.3390/biomedicines12061251

**Published:** 2024-06-04

**Authors:** Xiongjie Fu, Ming Wang, Yingfeng Wan, Ya Hua, Richard F. Keep, Guohua Xi

**Affiliations:** 1 Department of Neurosurgery, University of Michigan, Ann Arbor, MI 48109, USA; 2 Department of Neurosurgery, The 2nd Affiliated Hospital, Zhejiang University, Hangzhou 310027, China

**Keywords:** intracerebral hemorrhage, multinucleated giant cells, complement C3, phagocyte, hematoma clearance

## Abstract

Hematoma clearance is critical for mitigating intracerebral hemorrhage (ICH)-induced brain injury. Multinucleated giant cells (MGCs), a type of phagocyte, and the complement system may play a pivotal role in hematoma resolution, but whether the complement system regulates MGC formation after ICH remains unclear. The current study investigated the following: (1) the characteristics of MGC formation after ICH, (2) whether it was impacted by complement C3 deficiency in mice and (3) whether it also influenced hematoma degradation (hemosiderin formation). Young and aged male mice, young female mice and C3-deficient and -sufficient mice received a 30 μL injection of autologous whole blood into the right basal ganglia. Brain histology and immunohistochemistry were used to examine MGC formation on days 3 and 7. Hemosiderin deposition was examined by autofluorescence on day 28. Following ICH, MGCs were predominantly located in the peri-hematoma region exhibiting multiple nuclei and containing red blood cells or their metabolites. Aging was associated with a decrease in MGC formation after ICH, while sex showed no discernible effect. C3 deficiency reduced MGC formation and reduced hemosiderin formation. Peri-hematomal MGCs may play an important role in hematoma resolution. Understanding how aging and complement C3 impact MGCs may provide important insights into how to regulate hematoma resolution.

## 1. Introduction

Intracerebral hemorrhage (ICH) represents a common severe subtype of stroke with high morbidity and mortality rates, needing effective treatments [1]. The rapid influx of blood into brain tissue during ICH results in hematoma formation and the subsequent compression of surrounding parenchyma, causing primary mechanical damage. Over time, the release of hematoma metabolites, including hemoglobin, thrombin and peroxiredoxin 2, triggers neuroinflammation and neuronal death, contributing to secondary brain damage [2]. Therefore, methods to promote hematoma clearance may alleviate ICH-induced brain injury. In a number of clinical trials, surgical hematoma evacuation did not significantly improve neurological outcomes [3,4], but the recent ENRICH trial did show some benefit [5].

Another approach to promote hematoma clearance is via endogenous mechanisms. Intrinsic hematoma clearance, involving phagocytosis to reduce hematoma volume and eliminate erythrocytes and their metabolites, holds promise for attenuating ICH-induced secondary brain injury [6,7]. Macrophages and brain-resident microglia are key phagocytes involved in hematoma clearance [8]. Multinucleated giant cells (MGCs) are a special type of phagocyte formed by the cell–cell fusion of macrophages, and they have been identified as potential targets for promoting hematoma clearance [9]. Previous findings indicate that treatment with a CD47-blocking antibody enhances hematoma clearance, improves outcomes and increases MGC numbers in the peri-hematoma zone [9,10,11]. However, the mechanisms regulating MGC formation after ICH remain unclear.

Complement activation has many roles in ICH-induced brain injury including regulating inflammation, phagocytosis and cell lysis [12]. In vitro, Milde et al. [13] found that MGCs are specialized for the phagocytosis of complement-opsonized particles including erythrocytes where complement receptor 3 plays a key role. Moreover, in retinal diseases, C3 deficiency has been shown to reduce the phagocytosis by microglia [14]. In Alzheimer’s disease, the inhibition of C3 has been linked to a reduction in the number of phagocytic microglia [15]. Complement C3 is believed to play a crucial role in regulating phagocytosis. However, the specific role of the complement in MGC formation and hematoma clearance after ICH is not fully understood. The current study aimed to achieve the following: (1) Elucidate factors (aging/sex) influencing MGC formation after ICH and MGC characteristics. (2) Investigate whether complement C3 deficiency influences MGC formation after ICH. (3) Assess whether C3 deficiency also impacts the metabolism of the hematoma by examining hemosiderin deposition after ICH in C3-sufficient and -deficient mice.

## 2. Materials and Methods

### 2.1. Animal and ICH Model

The animal surgery protocol for this experiment was approved by the University of Michigan Committee on Use and Care of Animals. Surgeries were conducted following the Animal Research: Reporting of In vivo Experiments (ARRIVE) guidelines [16]. A total of thirty wild-type (WT) young male mice (C57BL/6J, The Jackson Laboratory, strain No. 000664), six WT young female mice, twelve WT aged male mice and eighteen C57BL/6J C3-deficient (The Jackson Laboratory, strain No. 029661) young mice were used to establish the autologous blood injection model, as previously described [10]. Briefly, mice were intraperitoneally anesthetized using ketamine (80 mg/kg) and xylazine (5 mg/kg), with subcutaneous administration and carprofen (5 mg/kg) for analgesia. A feedback-heated blanket maintained the core body temperature at approximately 37 °C. In the autologous blood injection model, whole blood was collected from the right femoral artery, and mice were secured in a stereotaxic frame from Kopf Instruments (Tujunga, CA, USA). Following sterilization, a 5 mm midline scalp incision and a 1 mm skull burr hole (0.6 mm anterior and 2.5 mm lateral to bregma) were made. Thirty microliters of autologous blood was injected into the right basal ganglia using a 26-gauge needle through the burr hole (3.5 mm depth, 3 μL/min). A micro-infusion pump from Harvard Apparatus Inc. performed the infusion, and the needle was left in place for 5 min to prevent reflux after completion. Random and even numbers were utilized for randomization, and no mice died during this project. Mouse brains were harvested on day 3 and day 7 after ICH.

### 2.2. Experimental Groups

This study comprises three main parts. In the first part, WT young male mice, WT young female mice and WT aged male mice received an injection of 30 μL autologous blood into the right basal ganglia. These ICH mice were euthanized on days 3 and 7. In the second part, WT young male mice and C3-deficient young male mice received an injection of 30 μL autologous blood into the right basal ganglia, and mice were euthanized on days 3 and 7. In the third part, WT young male mice and C3-deficient young male mice received an injection of 30 μL autologous blood into the right basal ganglia, and mice were euthanized on day 28. All brains were harvested for histology.

### 2.3. Hematoxylin and Eosin Staining

Mice were euthanized with a lethal dose of pentobarbital (100 mg/kg; I.P.) before trans-cardiac perfusion with 4% ice-cold paraformaldehyde (PFA). Brains were harvested and immersed in 4% PFA for 24 h before transferring to 30% sucrose at 4 °C until sinking. Brains were embedded in optimal cutting temperature (OCT) compound and 18 μm coronal sections taken on a cryostat for Hematoxylin and Eosin (H&E) staining using a protocol described previously [11].

### 2.4. Immunohistochemistry

Immunohistochemistry staining followed a previously described protocol [9]. Brain sections were dried and then immersed in 0.3% Triton X-100 for 15 min, followed by incubation with 10% goat serum for 1 h at room temperature. Brain slices were incubated overnight with primary antibodies: rat anti-MOMA-2 (monocyte + macrophage, Abcam, Boston, MA, USA, ab33451, 1:200) and rabbit anti-heme oxygenase-1 (HO-1, Enzo, SPA-895-F, 1:500) at 4 °C. After washing with methanol and H_2_O_2_, brain sections were incubated with the secondary antibodies: goat anti-rabbit IgG, Biotin (Invitrogen, Carlsbad, CA, USA, #31820, 1:500) and goat anti-rat IgG, Biotinylated (Vector Laboratories, Newark, CA, USA, BA-9400, 1:500) at room temperature for 90 min. After washing with PBS, brain sections were stained with stable DAB (Invitrogen, Carlsbad, CA, USA, #750118). Finally, slices were dehydrated, defatted, permeabilized and fixed with alcohol and xylene under different concentrations.

### 2.5. Immunofluorescence

Immunofluorescence staining was performed using a previously described protocol [9]. Brain sections were dried before being immersed in 0.3% Triton X-100 for 15 min. Sections were then incubated with 15% donkey serum for 1 h at room temperature. After washing with PBS, sections were incubated overnight with primary antibodies at 4 °C: rat anti-MOMA-2 (Novus Biologicals, Centennial, CO, USA, NB100-64946, 1:10), rabbit anti-HO-1 (Enzo, Farmingdale, NY, USA, SPA-895-F, 1:500), rabbit anti-complement receptor 3 (CR3) (Novus Biologicals, Centennial, CO, USA, NB100-89474, 1:200), and rat anti-CR3 (Abcam, Boston, MA, USA, ab8878, 1:50). After washing with PBS, sections were incubated with secondary antibodies: donkey anti-rabbit IgG, Alexa Flour 448 (Invitrogen, Carlsbad, CA, USA, A-21206, 1:500), donkey anti-rabbit IgG, Alexa Flour 594 (Invitrogen, Carlsbad, CA, USA, A-21207, 1:500), donkey anti-rat IgG, Alexa Flour 488 (Invitrogen, Carlsbad, CA, USA, A-21208, 1:500), and donkey anti-rat IgG, Alexa Flour 594 (Invitrogen, Carlsbad, CA, USA, A-21209, 1:500) at room temperature for 2 h. Finally, there sections were stained with DAPI (Sigma, Saint Louis, MO, USA, F6057) and observed under a fluorescence microscope.

### 2.6. Cell Counting

This process was conducted in a blinded manner. Three brain slices were reviewed per mouse, with each section examined in three different areas around the hematoma region. The numbers of target cells were determined using ImageJ software (NIH, Bethesda, MD, USA, version 1.5). Two approaches were employed to quantify changes in the number of MGCs. Firstly, the percentage of MGCs relative to all macrophages (MOMA-2+) was determined. MOMA-2 serves as a specific maker for macrophages, and all MGCs are MOMA-2-positive. However, not all MOMA-2-positive cells are MGCs. Therefore, utilizing a percentage calculation provides a representation of changes in MGCs. Secondly, the absolute number of MGCs positive for HO-1 was quantified. HO-1 is not a specific marker for phagocytes as it can be expressed in other cell types. Thus, we calculated the number of MGCs within the HO-1-positive cell population.

### 2.7. Statistical Analysis

All data are presented as means ± standard deviation (SD). A Shapiro–Wilk test was used to assess the normality of data distribution. Unpaired Student’s *t*-tests were applied for data with a normal distribution, while Mann–Whitney U-tests were applied for data with non-normal distribution. A *p*-value < 0.05 was considered statistically significant. Statistical analyses were performed using GraphPad Prism 8.0 (GraphPad Prism Software Inc., San Diego, CA, USA).

## 3. Results

### 3.1. MGCs: Location and Morphology after ICH

**Figure 1:**
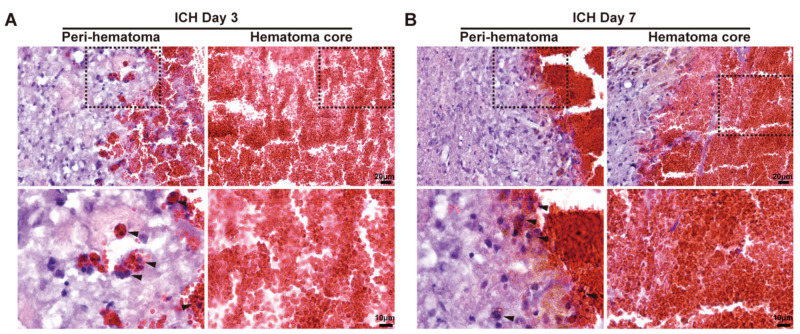
The location and morphology of MGCs after ICH on day 3 and day 7. (**A**) A representative H&E staining image of MGCs of WT male mice in the peri-hematoma and hematoma core region on day 3 after ICH. (**B**) A representative H&E staining image of MGCs of WT male mice in the peri-hematoma and hematoma core region on day 7 after ICH. The upper row scale bar = 20 μm (×40 objective), and the lower row scale bar = 10 μm (×100 objective). The black dashed line frame indicates the location of high-magnification viewing, and the black arrows represent the MGCs.

In young male mice, H&E staining revealed many large multinucleated cells immediately around the hematoma on days 3 and 7 after ICH (Figure 1). These MGCs often contained erythrocytes, and they were predominantly located in the peri-hematoma region rather than the hematoma core (Figure 1A,B).

### 3.2. The Aging-Induced Decrease in the Number of MGCs in the Peri-Hematoma Area

**Figure 2:**
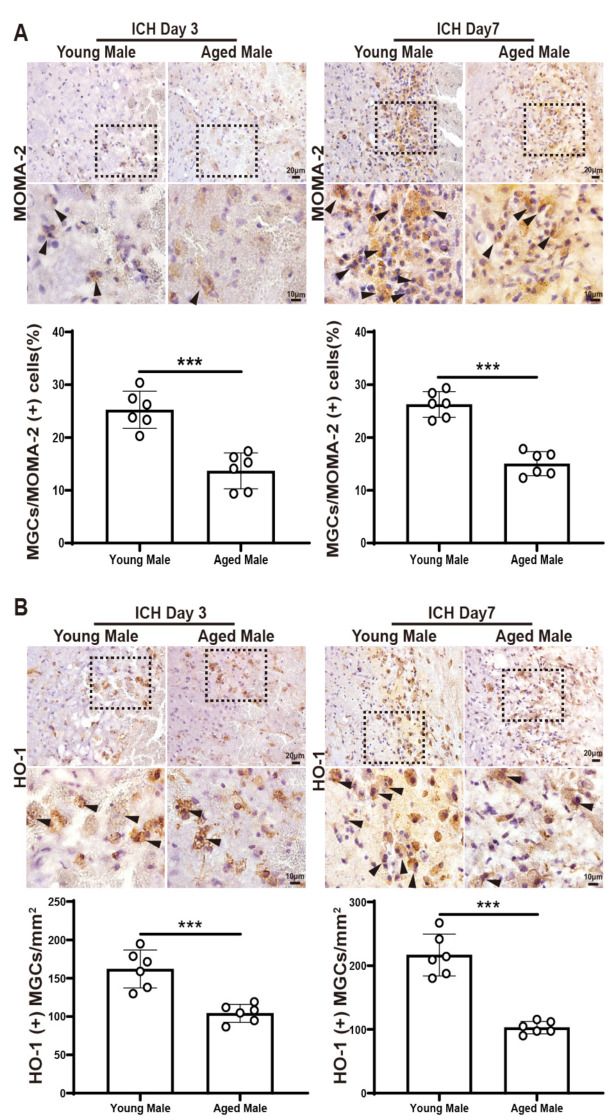
Aging induced a decrease in the number of MGCs in the peri-hematoma area in male mice on day 3 and day 7 after ICH. (**A**) A representative immunohistochemistry staining image of MOMA-2 in young and aged WT male mice in the peri-hematoma region on day 3 and day 7 after ICH. The quantification of the percentage of MOMA-2+ MGCs relative to total MOMA-2+ cells (*n* = 6 per group). (**B**) Representative HO-1 immunohistochemistry in young and aged WT male mice in the peri-hematoma region on day 3 and day 7 after ICH. The quantification of the number of HO-1+ MGCs (*n* = 6 per group). The upper row scale bar = 20 μm (×40 objective), and the lower row scale bar = 10 μm (×100 objective). The black dashed line frame indicates the location of high-magnification viewing, and the black arrows represent the MGCs. Data are expressed as the mean ± SD. *** *p* < 0.001.

To investigate the influence of age on MGC formation after ICH, two approaches were used. First, the number of MGCs as a percentage of all macrophages (MOMA-2+) in the peri-hematomal area was determined. Compared to young male adult mice, the percentage of MOMA-2+ MGCs significantly decreased in the peri-hematoma region in aged mice on day 3 (25.3 ± 3.5% vs. 13.7 ± 3.4%, *p* < 0.001, *n* = 6 per group) and day 7 (26.3 ± 2.4% vs. 15.0 ± 2.3%, *p* < 0.001, *n* = 6 per group) after ICH (Figure 2A). Secondly, the number of MGCs positive for HO-1 was determined. Again, compared to young adult mice, the number of HO-1+ MGCs showed a significant decrease in the peri-hematoma region in aged mice on day 3 (162 ± 25 vs. 104 ± 12 cells/mm^2^, *p* < 0.001, *n* = 6 per group) and day 7 (217 ± 33 vs. 103 ± 10 cells/mm^2^, *p* < 0.001, *n* = 6 per group) after ICH (Figure 2B).

In comparison, no significant differences were found in the percentage of MOMA-2+ MGCs and the number of HO-1+ MGCs in the peri-hematoma region between male and female young mice after ICH on day 3 (MOMA2: 25.3 ± 3.5% vs. 28.4 ± 2.8%, *p* = 0.1225, *n* = 6 per group, HO-1: 162 ± 25 vs. 169 ± 26 cells/mm^2^, *p* = 0.1282, *n* = 6 per group) (Supplementary Figure S1A,B).

### 3.3. C3-Deficient Mice Exhibited Significantly Fewer MOMA2-Positive MCGs in the Peri-Hematoma Area

**Figure 3:**
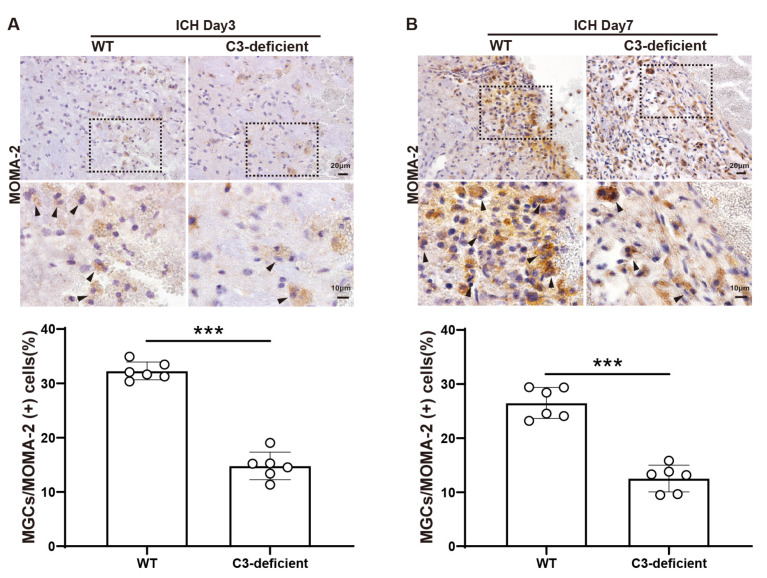
C3 deficiency decreases MGCs as a percentage of all MOMA-2+ in the peri-hematoma region in male mice on days 3 and 7 after ICH. (**A**) Representative MOMA-2 immunohistochemistry in WT and C3-deficient male mice in the peri-hematoma region on day 3 after ICH. The quantification of MOMA-2+ MGCs as a percentage of all MOMA-2+ cells in the two groups (*n* = 6 per group). (**B**) Representative MOMA-2 immunohistochemistry in wild-type and C3-deficient male mice in the peri-hematoma region on day 7 after ICH. The quantification of MOMA-2+ MGCs as a percentage of all MOMA-2+ cells in the two groups (*n* = 6 per group). The low magnification scale bar = 20 μm (×40 objective), and the high magnification scale bar = 10 μm (×100 objective). The black dashed line frame indicates the location of high-magnification viewing, and the black arrows represent the MOMA-2+ multinucleated giant cells. Data are expressed as means ± SD. *** *p* < 0.001.

Whether complement C3 is involved in MGC formation after ICH was examined. Immunofluorescence co-staining showed that MOMA-2+ or HO-1+ MGCs are complement receptor 3 (CR3)+ cells (Supplementary Figure S2A,B). In WT and C3-deficient mice, MOMA-2 staining revealed a significantly low percentage of MOMA-2+ MGCs in the peri-hematoma region in C3-deficient mice on day 3 (32.3 ± 1.6% in WT vs. 14.8 ± 2.5% in C3-deficient mice, *p* < 0.001, *n* = 6 per group) and day 7 (26.5 ± 2.9% in WT vs. 12.5 ± 2.5% in C3-deficient mice, *p* < 0.001, *n* = 6 per group) (Figure 3A,B).

### 3.4. C3-Deficient Mice Exhibited Significantly Fewer HO-1-Positive MCGs around the Hematoma

**Figure 4:**
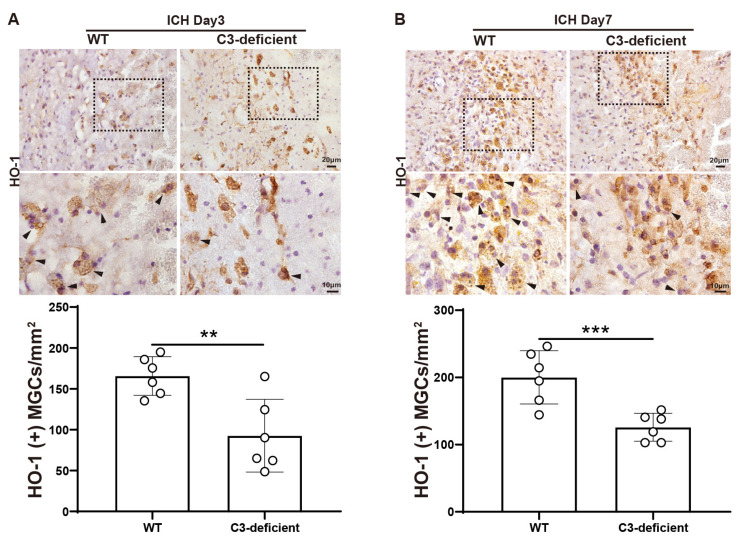
C3 deficiency decreases the number of HO-1+ MGCs in male mice on days 3 and 7 after ICH. (**A**) Representative peri-hematomal HO-1 immunohistochemistry in WT and C3-deficient mice on day 3 after ICH. The quantification of the number of HO-1+ MGCs. (**B**) Representative HO-1 immunohistochemistry in WT and C3-deficient mice in the peri-hematoma region on day 7 after ICH. The quantification of the number of HO-1+ MGCs. Lower and higher magnification scale bars = 20 μm (×40 objective) and 10 μm (×100 objective), respectively. The black dashed frame indicates the location of high-magnification viewing. Black arrows indicate HO-1+ multinucleated giant cells. Data are means ± SD, *n* = 6. ** *p* < 0.01.*** *p* < 0.001.

HO-1 staining also confirmed the effect of C3 deficiency on the formation of MGCs after ICH. C3-deficient mice had a significantly lower number of HO-1+ MGCs in the peri-hematoma region on day 3 (166 ± 24 cells/mm^2^ in WT vs. 93 ± 45 cells/mm^2^ in C3-deficient mice, *p* < 0.001, *n* = 6 per group) and day 7 (200 ± 40 cells/mm^2^ in WT vs. 126 ± 21 cells/mm^2^ in C3-deficient mice, *p* < 0.001, *n* = 6 per group) (Figure 4A,B).

### 3.5. C3-Deficient Mice Exhibited Reduced Hemosiderin Deposition in the Hematoma Region

**Figure 5:**
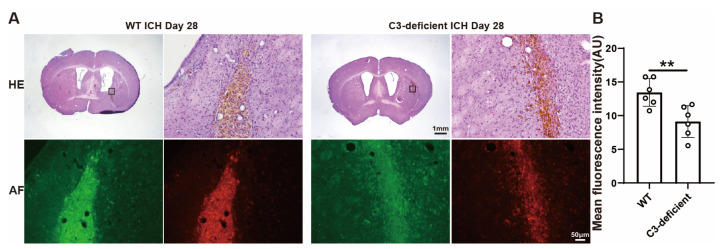
Hemosiderin deposition in the hematoma region on day 28 after ICH in wild-type and C3-deficient mice. (**A**) Representative H&E staining and autofluorescence (AR) images in WT and C3-deficient male mice in the hematoma region on day 28 after ICH (*n* = 6 per group). (**B**) The quantification of the mean autofluorescence intensity in WT and C3-deficient male mice in the hematoma region on day 28 after ICH. Lower and higher magnification scale bars = 1 mm (×1.25 objective) and 50 μm (×20 objective). The black frame is the location of high-magnification viewing. Data are expressed as the mean ± SD. ** *p* < 0.01.

Hemosiderin is a crucial byproduct of hematoma degradation after phagocyte metabolism. It was mainly located at the hematoma site and partially in the peri-hematoma region. Whether C3 deficiency impacts hemosiderin deposition after ICH was examined. Hemosiderin is autofluorescent, and fluorescence intensity was measured to evaluate hemosiderin deposition 28 days after ICH. C3-deficient mice had a lower mean hemosiderin fluorescence intensity in the hematoma core compared to WT mice (13.5 ± 2.1 Arbitrary Units (AU) in WT mice vs. 9.1 ± 2.4 AU in C3-deficient mice, *p* < 0.001, *n* = 6 per group) (Figure 5A,B). This suggests a potential association between C3 deficiency, reduced MGC formation and reduced hemosiderin deposition after ICH.

## 4. Discussion

This study elucidates the characteristics and role of C3 in MGC formation after ICH in mice. Our observations indicate that MGCs are primarily located in the peri-hematoma region after ICH and that age but not sex significantly influenced their formation. Secondly, C3 emerges as a key factor in MGC formation, with C3 deficiency leading to decreased numbers of MGCs. Thirdly, C3 deficiency decreases hemosiderin deposition at the site of the hematoma which may reflect the role of C3 in MGC formation and thus hemoglobin degradation.

MGCs are a specialized type of macrophages formed through the fusion of single mononuclear cells. They play a crucial role in various conditions, including cancer, metabolic disorders, infection and nervous system diseases [17,18,19,20]. MGCs play a crucial role in tissue remodeling and maintaining homeostasis, with phagocytosis being a key function [21]. After ICH, hematoma clearance is vital for mitigating ICH-induced brain injury [22]. For example, previous research has demonstrated that treatment with a CD47-blocking antibody, to inhibit a ‘don’t-eat-me’ signal, enhances hematoma clearance and alleviates brain injury in mice [10]. After treatment, the number of MGCs around the hematoma increased [9,23]. Previous investigations have also demonstrated a pronounced increase in macrophage phagocytic activity on days 3 and 7 post-ICH [10]. Concurrently, there is a notable abundance of MGCs at these time points [9]. Consequently, in the present study, we selected days 3 and 7 post-ICH to conduct our observations of MGCs. MGCs can be found with multiple engulfed erythrocytes, and these results suggest that MGCs play a pivotal role in hematoma clearance after ICH. Therefore, understanding the characteristics and the factors influencing MGC formation after ICH could be crucial for advancing treatment.

Aging is a common factor that influences cell and tissue function as well as homeostasis. In the normal aging process, the phagocytic capacity of phagocytes tends to decline [24]. However, in neurodegeneration diseases, phagocytosis by microglia increases with aging [25]. The impact of aging appears to vary under different conditions. In the present study, aging was correlated with a reduction in the number of MGCs around the hematoma. Although the detailed effect of aging on hematoma clearance remains unclear, our results suggest that aging might decelerate hematoma clearance. The precise mechanisms require further research in the future. Regarding sex, our observations indicate that there is no significant difference in the number of MGCs around the hematoma between male and female mice.

The complement system is a key component of the immune system, regulating tissue function and homeostasis [26,27]. C3, an essential complement component, is implicated in various diseases, including autoimmune disease, cancer, kidney diseases, metabolic diseases and neurological diseases [28,29,30,31,32]. Its diverse functions vary across different disease contexts. One crucial role of C3 is to regulate phagocytosis [14]. Hematoma clearance has emerged as a pivotal process for mitigating ICH-induced brain injury, and MGCs may be a key contributor to this clearance [18]. Both in vivo and in vitro studies have indicated that the phagocytic capacity of MGCs is dependent on complement [13].

CR3, a receptor of C3, is important in the activation of MGCs [13]. In the present study, we observed that most MGCs are CR3-positive, leading us to speculate that C3 is intricately involved in the formation of MGCs following ICH. Notably, our findings reveal a diminished presence of MGCs in the peri-hematoma region in C3-deficient mice, suggesting a potential slowdown in the rate of hematoma clearance (as suggested by reduced hemosiderin formation in those mice). Previous studies have suggested that C3 deficiency can reduce erythrolysis within the hematoma and alleviate ICH-induced brain injury, indicating that C3 mediates multiple functions in the pathophysiological process of ICH [33]. The benefits arising from the reduction in erythrolysis due to C3 deficiency appear to outweigh the drawbacks associated with the decrease in the number of MCGs. Achieving a balance in C3 regulation to simultaneously decrease erythrolysis while increasing the number of MGCs may further reduce brain damage caused by ICH. Hematoma resolution is increasingly recognized as a critical factor in reducing brain damage following ICH, with MGCs playing a significant role in this process. Our research indicates that complement C3 is crucial for the formation of MGCs around the hematoma. Therefore, targeting C3 could potentially be a therapeutic strategy for patients with ICH either alone or in combination with surgical evacuation [5].

The number of phagocytes and their phagocytic ability are crucial factors for hematoma clearance. In this study, we explored the hemosiderin deposition as an end product of hematoma phagocytosis. Hemosiderin is an important byproduct of hemoglobin metabolism. It forms and deposits within cells through a series of oxidative reactions after erythrocyte phagocytosis [34]. Hemosiderin exhibits autofluorescence [35], and we measured the fluorescence intensity to assess hemosiderin deposition. At the site of the hematoma, the autofluorescence intensity decreases in C3-deficient mice. We hypothesize that C3 deficiency leads to a decrease in the phagocytic capacity of MGCs resulting in less hemosiderin deposition. Whether this reflects a decline in the phagocytic ability of individual MGCs, or the total number of MGCs or both requires further study.

While the present study identified age and C3 as key factors regulating MGC formation, it has some limitations: (1) Although MOMA-2 and HO-1 are commonly used markers for MCGs, they are not specific indicators of MGCs. (2) The detailed mechanism of C3 regulating the formation of MGCs and their phagocytic activity after ICH has not been elucidated. Therefore, further studies are needed. (3) We have not compared hematoma volumes in C3-deficient and WT mice after ICH.

## 5. Conclusions

**Figure 6:**
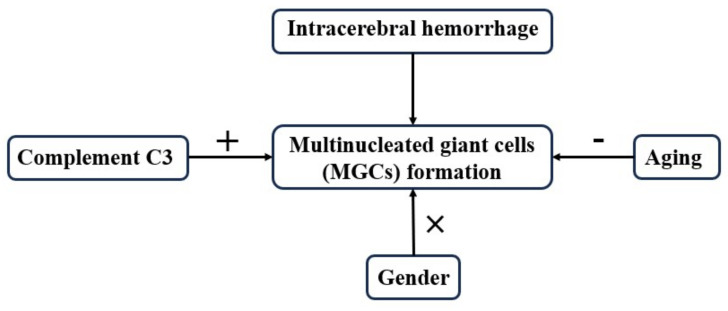
Intracerebral hemorrhage induced the formation of multinucleated giant cells (MGCs). Aging decreased (−) the number of MGCs in the peri-hematoma area. Gender had no impact (X) on MGC formation around the hematoma. Complement C3 enhanced (+) the number of MCGs in the area surrounding the hematoma.

In conclusion, our study explores the characteristics of MGC formation after ICH and the potential role of complement C3 (Figure 6). The findings reveal that age and C3 deficiency both impact ICH-induced MGC formation which may then influence hematoma clearance and hemoglobin degradation (hemosiderin production). Targeting MGCs could be a potential treatment strategy for promoting hematoma clearance after ICH.

## Data Availability

All supporting data are contained within the article. The datasets used and/or analyzed in this study are available from the corresponding authors upon reasonable request.

## Supplementary Materials

The following supporting information can be downloaded at https://www.mdpi.com/article/10.3390/biomedicines12061251/s1, Figure S1: The number of multinucleated giant cells in the peri-hematoma region on day 3 after ICH in WT male and female mice; Figure S2: MOMA-2-positive MGCs are CR3-positive cells.
