# Supplementary material for: Formation of Multinucleated Giant Cells after Experimental Intracerebral Hemorrhage: Characteristics and Role of Complement C3
Source: Biomedicines. 2024 Jun 4;12(6):1251. doi: 10.3390/biomedicines12061251 (PMC11201741; doi:10.3390/biomedicines12061251)
Supplement: Supplementary file 1 [file biomedicines-12-01251-s001.zip › biomedicines-3006690-supplementary.pdf]

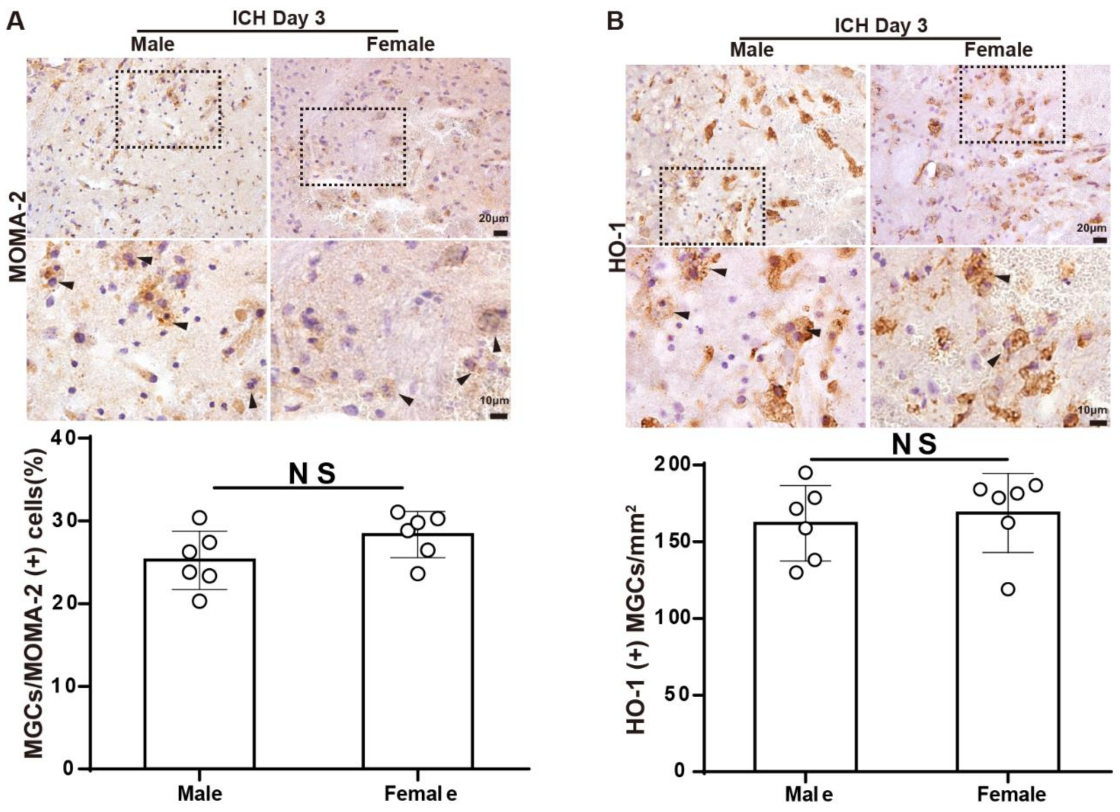

Figure S1. No difference in the number of multinucleated giant cells (MGCs) in the peri-hematoma region on day 3 after ICH between WT male and female mice. **A** Representative MOMA-2 immunohistochemistry images in male and female mice in the peri-hematoma region on day 3 after ICH. MOMA-2^+ MGCs as a percentage of all MOMA-2^+ cells 3 days after ICH (n = 6 per group). **B** Representative HO-1 immunohistochemistry images in male and female mice in the peri-hematoma region on day 3 after ICH. Quantification of the number of HO-1^+ MGCs 3 days after ICH (n = 6 per group). The low magnification scale bar = 20 μm (×40 objective), and the high magnification scale bar = 10 μm (×100 objective). The black dotted frame indicates the location of high magnification viewing. Data are means ± SD. NS = not significant.

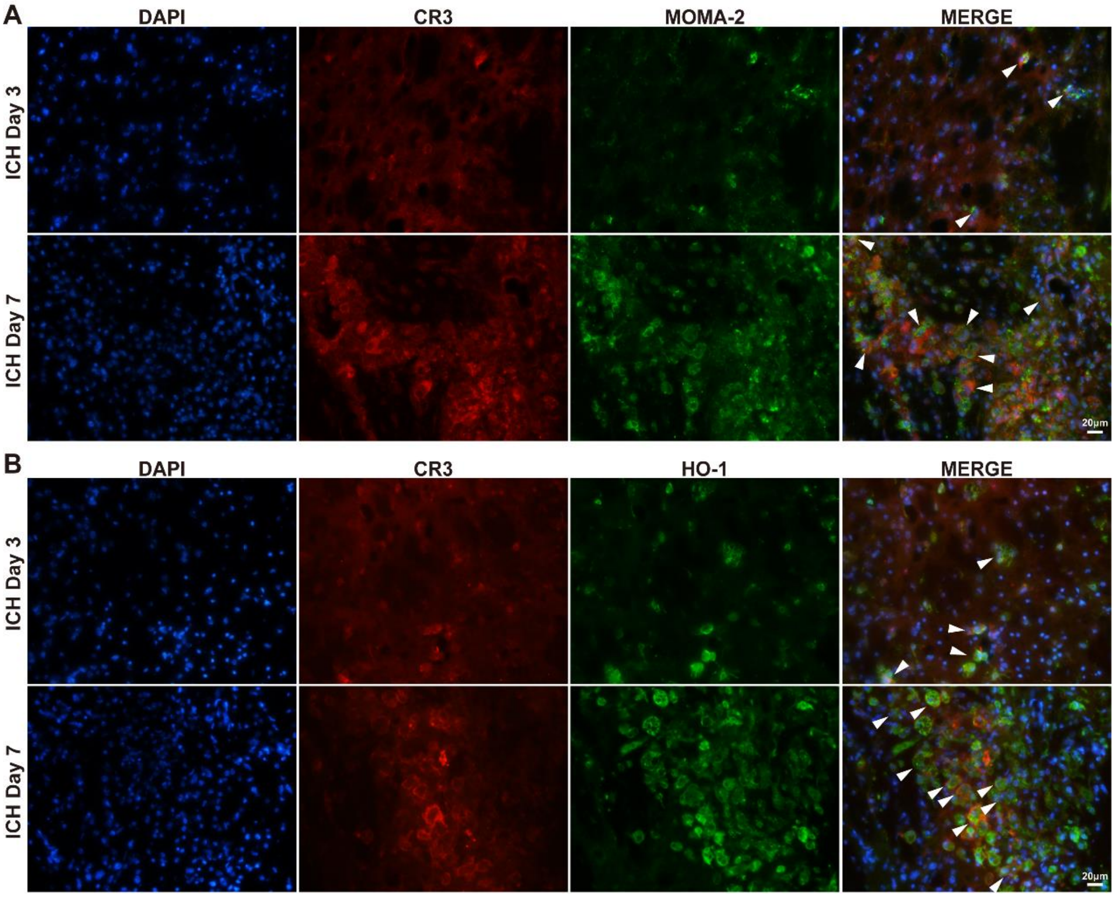

Figure S2. Colocalization of CR3 immunofluorescence with MOMA-2 positive and HO-1 positive MGCs. **A** Representative immunofluorescence images of CR3 and MOMA-2 in WT male mice in the peri-hematoma region on days 3 and 7 after ICH. **B** Representative immunofluorescence images of CR3 and HO-1 in WT male mice in the peri-hematoma region on days 3 and 7 after ICH. DAPI was used as a nuclear counterstain. Scale bars = 20 μm (×40 objective). The white arrows represent the double positive MGCs.
